# Changes of epicardial fat thickness after laparoscopic sleeve gastrectomy: a prospective study

**DOI:** 10.1080/07853890.2021.1903072

**Published:** 2021-05-19

**Authors:** Ahmed Abdallah Salman, Mohamed Abdalla Salman, Ahmed Soliman, Ahmed Youssef, Safa Labib, Mona Youssry Helmy, Mohamed A. Marie, Mohamed Shawkat, Amir Mostafa, Mohamed Sabry Tourky, Mohamed D. Sarhan, Mohamed Gamal Qassem, Hossam El-Din Shaaban, Mahmoud Gouda Omar, Tarek Elsayed Abouelregal

**Affiliations:** a Internal Medicine Department, Faculty of Medicine, Cairo University, Giza, Egypt; b General Surgery Department, Faculty of Medicine, Cairo University, Giza, Egypt; c Cardiovascular Department, Faculty of Medicine, Cairo University, Giza, Egypt; d Department of Surgery, Great Western Hospitals NHS Foundation Trust, Swindon, UK; e Department of General Surgery, Faculty of Medicine, Ain Shams University, Cairo, Egypt; f Gastroenterology Department, National Hepatology and Tropical Medicine Research Institute, Cairo, Egypt

**Keywords:** Bariatric surgery, laparoscopic sleeve gastrectomy, epicardial fat, atherosclerosis

## Abstract

**Purpose:**

Epicardial fat thickness is an interesting parameter of early atherosclerosis. We prospectively assessed whether weight loss following laparoscopic sleeve gastrectomy (LSG) leads to a significant reduction in the epicardial fat thickness (EFT) and the correlation between the decline in the epicardial fat thickness with other clinical parameters.

**Methods:**

A prospective analysis of 98 cases that were scheduled to undergo LSG and followed up for 12 months was conducted. EFT was assessed using two-dimensional (2 D) echocardiography.

**Results:**

A total of 98 cases and 70 controls were enrolled. EFT demonstrated a significant reduction at follow-up in the whole group (median 8.9 (1.95) versus 7.65 (1.67) mm, respectively). The degree of reduction was higher in the LSG cohort compared to control cohort 1.3 (0.4) versus 1 (0.4), respectively; *p* < .001). The univariate regression analysis demonstrated a notable correlation of the EFT with the weight, body mass index (BMI), fasting blood glucose (FBG), and creatinine with a *p*-value of <.0001, .001, .022, and .018, respectively while the multivariate analysis showed a strong correlation between EFT and weight and creatinine with a *p*-value of <.0001 and .033 respectively.

**Conclusion:**

LSG can have a favourable impact on metabolic syndrome aspects, namely EFT, as it can decrease it considerably.

## Introduction

Obesity is a global epidemic whose incidence has tripled in the past few decades; according to recent global figures by World Health Organisation (WHO), up to 650 adults suffer from obesity worldwide [1]. Morbid obesity is a severe form of the disease that is defined by a body mass index of more than 40Kg/m^2^ with a progressive increase in the hazard of mortality and morbidities [2]. Cases with morbid obesity are at an elevated risk of cardiovascular disorders (CVDs), thromboembolic events, diabetes mellitus (DM), non-alcoholic fatty liver disease, gastroesophageal reflux, chronic kidney disease, sleep apnoea, and carcinogenic events [2–4]. Additionally, morbid obesity is associated with a significant impairment in physical function, leading to limited daily activities, disability, and reduced quality of life [5].

Although diet modifications, lifestyle changes, and pharmacological interventions represent the first-line management approaches in the setting of obesity, the current body of evidence shows limited improvement and unsatisfactory results of these approaches in morbidly obese patients [6,7]. Metabolic procedures are the gold standard strategy for obese cases that are refractory to non-surgical management; laparoscopic sleeve gastrectomy (LSG) is a widespread restrictive, simple surgery that exhibited high efficacy and well-tolerable safety profile in the management of morbid obesity [8]. Previous reports demonstrated superior outcomes of LSG over non-surgical approaches regarding weight loss, survival, CVD mortality, and components of metabolic syndrome [9]. Moreover, patients who underwent LSG demonstrated a significant reduction in the parameters of the inflammatory response, such as C-reactive protein (CRP), cytokines, and adipocytokines [10].

On the other hand, regional fat distribution is a quite novel and independent parameter of increased risk of hypertension, dyslipidemia, and other CVDs risk factors in obese patients and the general population [11,12]. Epicardial fat is a visceral adipose tissue, which actively secretes free fatty acids, adipocytokines, and other pro-inflammatory cytokines [13]. According to recent reports, the progression of epicardial fat thickness is substantially linked to early atherosclerosis, coronary artery diseases, left ventricular hypertrophy, and metabolic syndrome [14,15]. Thus, the epicardial fat estimation can be viewed as an accurate indicator of CVDs in obese cases. Nonetheless, the impact of metabolic operations on the epicardial fat is understudied. Thus, we prospectively assessed whether weight loss following LSG leads to a significant reduction in the epicardial fat thickness and the correlation between the decline in the epicardial fat thickness with other clinical parameters.

## Materials and methods

A prospective cohort analysis was carried out on morbid obese cases (BMI ≥40kg/m^2^)that underwent LSG, after the failure of non-surgical approaches, in the period from March 2013 to February 2017. The decision for LSG was based on the criteria of the European guidelines on metabolic and bariatric surgery [16]. In addition, matched obese patients on a conservative management strategy were included as a control group. Patients with poorly controlled DM, a high-risk profile of CVDs, history of thromboembolic events, advanced heart failure, bleeding disorders, CKD, organ failure, malignancy, psychiatric disorders, mental illness, and/or active infection were excluded. In addition, we excluded patients who refused to sign the written informed consent.

### Data collection

After initial screening, all eligible patients were subjected to history taking and full physical examination. In addition, the following laboratory parameters were collected: lipid profile, blood glucose profile, glycated haemoglobin (HbA1c), homeostasis model assessment index for insulin resistance (HOMA-IR), liver function tests, and serum creatinine. A standard two-dimensional (2 D) echocardiographic evaluation was performed in all eligible patients as well. Cases were followed-up for twelve months after the operation.

### Laparoscopic sleeve gastrectomy surgery

The operation was performed through four trocar-port. We employed a Ligasure to split the greater omentum from the stomach. We placed a gastric calibration tube (36-Fr boggie) into the gastric lumen. Ultimately, we used a Covidien 3-0 V-Loc suture with seromuscular suturing to strengthen the whole staple line.

### 2D echocardiography and epicardial fat thickness (EFT)

**Figure 1.**
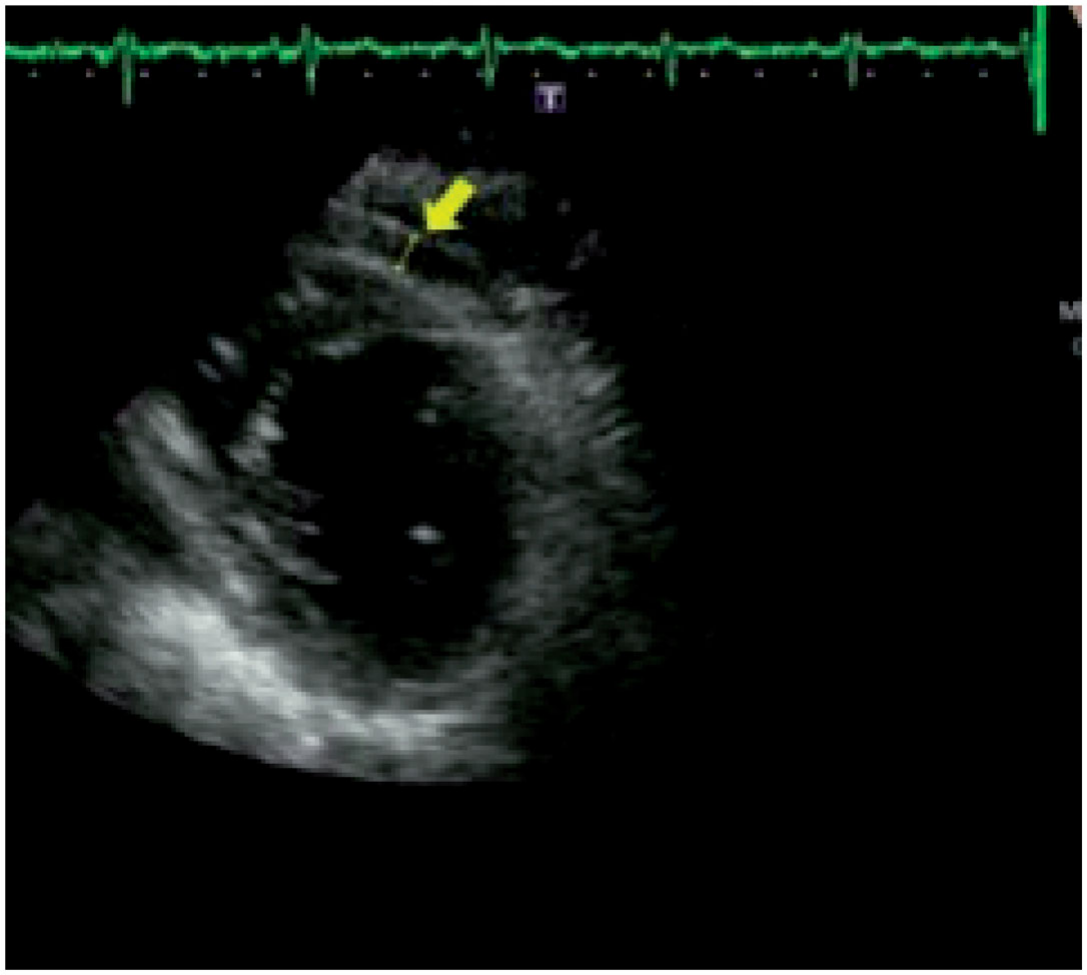
Epicardial fat thickness measurement.

The assessment was done by an expert physician who was blinded to the patient's information. Using Vivid S5 ultrasound machine (GE, Healthcare, Horten, Norway), we were able to get the apical and the conventional parasternal aspects. Also, this ultrasound was supplied by the transducer with 3SRS broadband. All the included patients underwent standard 2D and Doppler echocardiography. All procedures were encountered in compliance with the American Society of Echocardiography and the European Association of Cardiovascular Imaging (EACVI) guidelines [17]. The estimation of ejection fraction was based on the modified Simpson's method. The echo-free zone noticed between the myocardial free wall and pericardium visceral wall was considered the EFT (Figure 1).

EFT was assessed by standard 2D echocardiography in the parasternal aspect for three continuous cardiac cycles. Also, it was measured perpendicular to the right ventricular free wall [18].

### Study’s outcomes

The primary objective of this prospective work was to assess the changes in the epicardial fat thickness twelve months after LSG. At the same time, the secondary endpoints included the correlation between the changes in the epicardial fat thickness and the extent of weight reduction.

### Statistical methods

Statistical analysis was carried out using SPSS version 22. Categorical data were summarised using numbers and percentages. Continuous data were tested for normality using the Kolmogorov–Smirnov test. Normally distributed data were expressed in mean and standard deviations, while variables that were not normally distributed were expressed in median and interquartile range. A chi-squared test was used to compare the categorical variables. Hypothesis testing in the same group was conducted using Wilcoxon signed ranks; however, testing between separate groups was done using the Mann–Whitney *U* test. A *p*-value of less than .05 was considered significant, and the null hypothesis was rejected.

## Results

**Figure 2.**
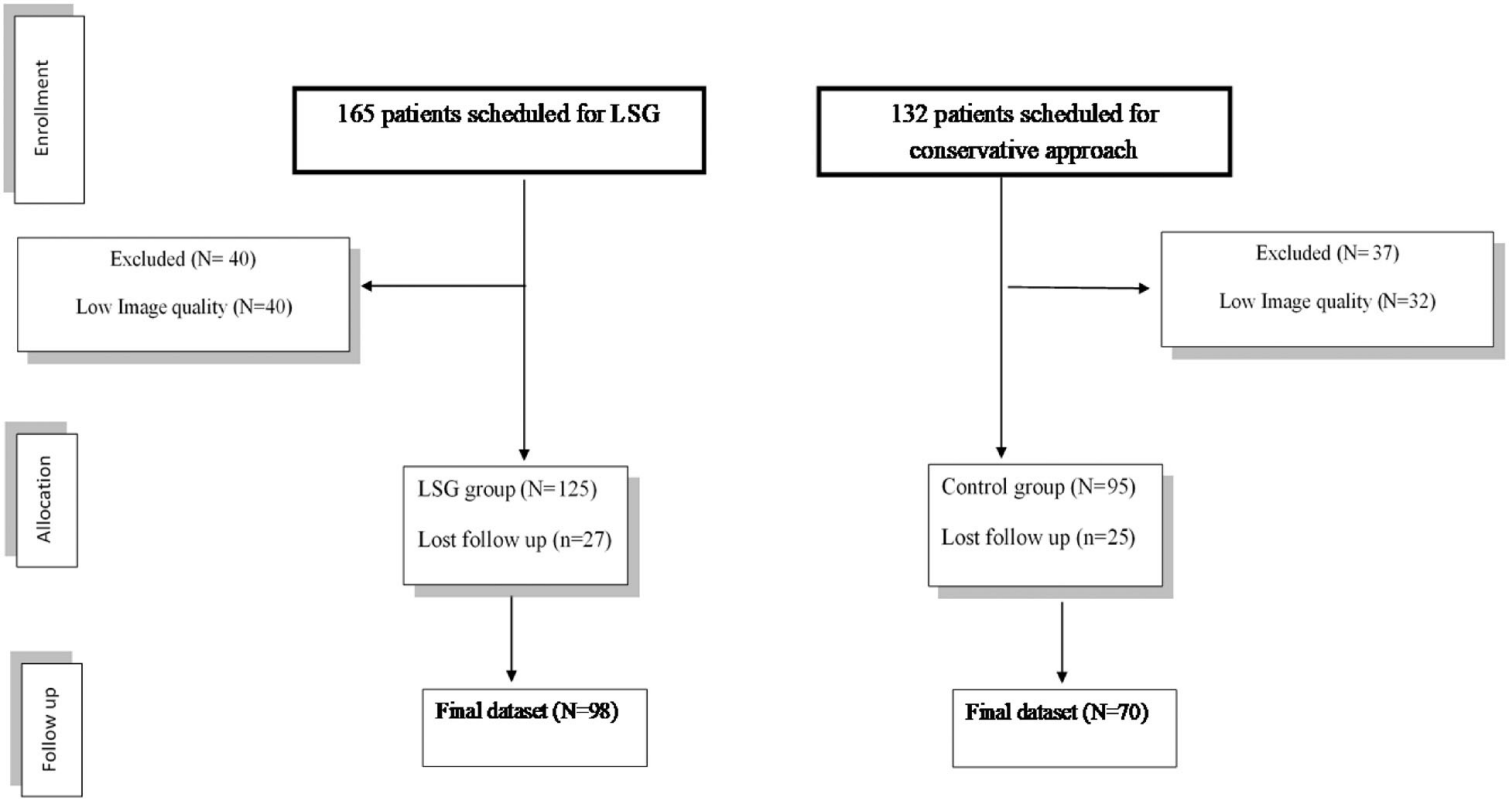
Patients who were enrolled in the final dataset analysis.

At the study's enrolment, 165 patients were screened; of them, 40 patients were excluded due to poor imaging quality, and 27 patients were lost during the follow-up period. Thus, a total of 98 subjects and 70 controls were enrolled in the final dataset analysis (Figure 2).

The median age of the included cases was 43 (11) years, compared to 41 (11) years in the control arm (*p* = .42). Both cases and control groups had comparable demographic, clinical, and laboratory parameters. However, the LSG group had significantly low LDL levels (*p* = .017) and higher HOMA-IR levels than the control groups (*p* < .001). In terms of echocardiographic findings at the baseline, both study groups had similar findings, except for septum thickness, which was higher in the control arm (*p* = .0.26; Table 1). There were some post-operative complications that included five staple line bleeding, two pulmonary embolisms, and one developed surgical site contamination. All of them were managed conservatively. There were no encountered mortalities within the study period.

**Table 1.** Characteristics of the both groups prior to intervention.

	Group 1 (LSG = 98)	Group 2 (Conservative = 70)	*p* Value
Age (years)			
Median (IQR)	43 (11)	41 (11)	.425
Gender (Number, %)			
Male	48 (49%)	42 (60%)	.158
Female	50 (51%)	28 (40%)	
Smoking (Number, %)	28 (28.6%)	17 (24.3%)	.539
FH of CAD (Number, %)	19 (19.4%)	14 (20%)	.922
Diabetes (Number, %)	28 (28.6%)	19 (27.1%)	.839
Hypertension (Number, %)	37 (37.8%)	21 (30%)	.297
Hyperlipidemia (Number, %)	21 (21.4%)	16 (22.9%)	.826
Antidiabetic agents (Number, %)	32 (32.7%)	25 (35.7%)	.679
Antihypertensive Agents (Number, %)	28 (28.6%)	23 (32.9%)	.551
Lipid lowering Agents (Number, %)	17 (17.3%)	14 (20%)	.662
mABP (mmHg, Median/IQR)	102 (18)	100.6 (15)	.190
FBG (mg/dl, Median/IQR)	117 (89)	138 (74)	.517
FBI (mIU/ml, Median/IQR)	21 (9.5)	23 (12)	.518
HBA1C (Median/IQR)	5.9 (1.3)	5.9 (1.3)	.518
Triglycerides (mg/dl, Median/IQR)	185 (47)	178 (68)	.322
T-Cholesterol (mg/dl, Median/IQR)	195 (85)	189.5 (76)	.582
LDL-C (mg/dl, Median/IQR)	101 (56)	122 (70)	.017
ALT (U/L, Mean/SD)	38.3 (6.6)	30.02 (5)	<.0001
AST (U/L, Median/IQR)	32 (7)	32 (5.8)	.158
Creatinine (mg/dl, Median/IQR)	0.9 (0.25)	1 (0.2)	.198
HOMA-IR (Mean /SD)	6.6 (1.5)	5.4 (1.6)	<.0001
Body Weight (kg, Mean /SD)	136.74 (11)	136.72 (11)	.993
BMI (kg/m^2^, Mean /SD)	44.7 (3.2)	44.4 (2.7)	.72
LAD (cm)			
Range	3–4.3	3–4.3	.621
Median (IQR)	3.6 (0.3)	3.6 (0.32)	
RAD (cm)			
Range	2.7–4.2	2.6–3.7	.269
Median (IQR)	3.2 (0.3)	3.2 (0.35)	
ESD (cm)			
Range	2.5–3.6	2.4–3.9	.253
Median (IQR)	3.2 (0.4)	3 (0.7)	
EDD (cm)			
Range	4.2–5.8	4–5.6	.286
Median (IQR)	5 (0.4)	4.9 (0.3)	
Septum Thickness (cm)			
Range	1–1.4	1–1.4	.026
Median (IQR)	1.2 (0.1)	1.2 (0.2)	
Post wall Thickness (cm)			
Range	1–1.33	1–1.3	.222
Median (IQR)	1.2 (0.1)	1.2 (0.1)	
EF (%)			
Range	51–69	53–70	.930
Median (IQR)	61 (7)	61 (7)	
EFT (mm)			
Range	6.6–10.9	6.9–10.6	.340
Median (IQR)	8.9 (1.95)	8.7 (1.9)	

mABP: mean arterial blood pressure; FBG: fasting blood sugar; FPI: fasting plasma insulin; LDL: low density lipoproteins; ALT: alanine transaminase; AST: aspartate transaminase BMI: body mass index, HOMA, LAD: left atrial diameter; RAD: right atrial diameter; ESD: end systolic diameter; EDD: end diastolic diameter; EF: ejection fraction; EFT: epicardial fat thickness.

In the 12th month after the operation, the LSG group had significantly lower values of body weight, BMI, mean arterial blood pressure, fasting blood glucose, fasting insulin, HbA1c, LDL, liver enzymes, and HOMA-IR (*p* > .05; Table 2). All patients had a significant drop in the left atrial diameter, end-systolic diameter, end-diastolic diameter, septum thickness, and post wall thickness. On comparing the echocardiographic parameters at the end of follow-up, patients in the LSG group had significantly lower septum thickness and ejection fraction than the control arm (*p* > .05; Table 2).

**Table 2.** Follow up of the metabolic status and echocardiographic findings of both groups post intervention.

	Group 1 (LSG = 98)	Group 2 (Conservative = 70)	*p* Value
Diabetes cured (Number, %)	16 (16.3%)	11 (15.7%)	.861
Hypertension cured (Number, %)	25 (25.5%)	11 (15.7%)	.127
Hyperlipidemia cured (Number, %)	13 (13.3%)	8 (11.4%)	.723
mABP (mmHg, Median/IQR)	89.8 (12)	94.3 (6.9)	<.0001
FBG (mg/dl, Median/IQR)	90 (19)	132 (76)	<.0001
FBI (mIU/ml, Median/IQR)	10 (6)	15 (7.5)	<.0001
HBA1C (Median/IQR)	5.3 (0.65)	5.7 (0.7)	.001
Triglycerides (mg/dl, Median/IQR)	145 (36)	152 (34)	.586
T-Cholesterol (mg/dl, Median/IQR)	157 (22)	89 (49)	.106
LDL-C (mg/dl, Median/IQR)	78 (17)	122 (70)	<.0001
ALT (U/L, Mean/SD)	23 (6)	28 (7.3)	<.0001
AST (U/L, Median/IQR)	26 (8)	30 (9)	<.0001
Creatinine (mg/dl, Median/IQR)	0.9 (0.2)	0.9 (0.1)	.133
HOMA-IR (Mean /SD)	2.7 (1.25)	4.3 (2)	<.0001
Body Weight (kg, Mean /SD)	98.9 (8.7)	122.4 (12.4)	<.0001
BMI (kg/m^2^, Mean /SD)	32.4 (3.4)	38.9 (3.2)	<.0001
Lost Weight (Kg, Median, IQR)	37.5 (16)	12.5 (5.4)	<.0001
LAD (cm)			
Range	3.1–4.1	3–4.37	.797
Median (IQR)	3.6 (0.3)	3.5 (0.3)	
RAD (cm)			
Range	2.7–4.2	2.7–3.6	.370
Median (IQR)	3.1 (0.3)	3.2 (0.4)	
ESD (cm)			
Range	2.6–3.5	2.4–3.9	.864
Median (IQR)	3 (0.35)	3 (0.52)	
EDD (cm)			
Range	4–5.7	4.1–5.5	.130
Median (IQR)	5 (0.3)	4.9 (0.33)	
Septum Thickness (cm)			
Range	1–1.4	1–1.4	<.0001
Median (IQR)	1.2 (0.2)	1.1 (0.1)	
Post wall Thickness (cm)			
Range	1–1.4	1–1.4	.194
Median (IQR)	1.1 (0.1)	1.1 (0.1)	
EF (%)			
Range	52–70	53–69	.005
Median (IQR)	62 (6)	60 (6.25)	
EFT (mm)			
Range	5–9.6	5.8–9.7	.390
Median (IQR)	7.7 (1.65)	7.65 (1.8)	
EFT Lost (mm)			
Range	0.1–1.8	0.6–1.6	<.0001
Median	1.3 (0.4)	1 (0.4)	

mABP: mean arterial blood pressure; FBG: fasting blood sugar; FPI: fasting plasma insulin; LDL: low density lipoproteins; ALT: alanine transaminase; AST: aspartate transaminase BMI: body mass index; HOMA, LAD: left atrial diameter; RAD: right atrial diameter; ESD: end systolic diameter; EDD: end diastolic diameter; EF: ejection fraction; EFT: epicardial fat thickness.

**Figure 3.**
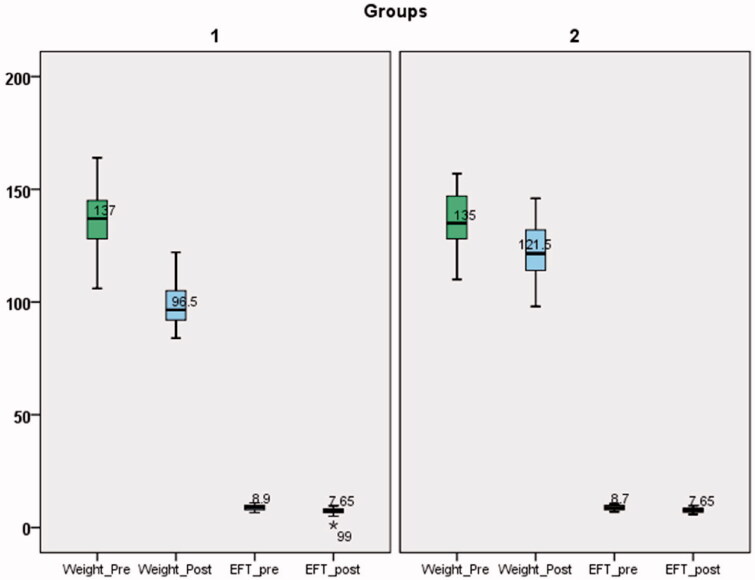
Box Plot demonstrating the weight and EFT change in both groups pre & post intervention.

In terms of the main perspective of the present work, the epicardial fat thickness demonstrated a significant reduction at the end of follow-up in the whole group (median 8.9 (1.95) versus 7.65 (1.67) mm, respectively). The degree of epicardial fat thickness reduction was higher in the LSG cohort compared to control cohort1.3 (0.4) versus 1 (0.4), respectively; *p* < .001; Figure 3).

The univariate regression analysis revealed a considerable correlation of the epicardial fat thickness with the weight, BMI, FBG, and creatinine with a *p*-value of <.0001, .001, .022, and .018 respectively while the multivariate analysis showed a strong correlation between epicardial fat thickness and weight and creatinine with a *p*-value of <.0001 and .033 respectively (Table 3).

**Table 3.** Univariate and multivariate regression model of EFT.

Univariate					Multivariate			
Variable	Beta coefficient	sig	95% confidence interval				95% confidence interval	
			Lower bound	Upper bound	Beta coefficient	sig	Lower bound	Upper bound
Weight	0.422	<0.0001	0.021	0.042	0.887	<0.0001	0.041	0.079
BMI	0.257	0.001	0.027	0.102				
FBG	0.177	0.022	0.001	0.009				
Creatinine	0.183	0.018	0.03	0.319	0.132	0.033	0.01	0.241

## Discussion

To date, only a few reports assessed the changes in the regional fat distribution following bariatric surgery and the correlation between these changes and other clinical parameters. Thus, we aimed to determine whether weight loss following LSG leads to a decrease in the epicardial fat thickness. We demonstrated that LSG led to a statistically significant reduction in the epicardial fat thickness twelve months after the operation. The extent of the decrease in the epicardial fat thickness was more notable following LSG than the conservative approach. In addition, the reduction in epicardial fat thickness correlated strongly with the degree of weight reduction at 12 months of follow-up and to a lesser degree with serum creatinine (incidence of coronary artery disease may increase with chronic kidney disease).

BMI is the universally accepted indicator of the degree and severity of obesity. However, it has been recently recognised that obesity is a heterogeneous disorder that exhibited variable degrees of adverse metabolic abnormalities, even in individuals with similar BMI [17]. Owing to these findings, a growing body of published literature aimed to characterise the critical role of visceral fat deposition in the development of obesity comorbidities [18]. Epicardial fat thickness is a quite novel marker for visceral fat deposition that is simply measured by routine 2 D echocardiography; the assessment of epicardial fat thickness depends on measuring the accumulated fat between the pericardium and the myocardium [14]. Previous reports showed that an increase in epicardial fat is linked to a higher risk of dyslipidemia, atherosclerosis, insulin resistance, coronary artery disease, and structural abnormalities of the heart [19,20]. The release of inflammatory cytokines and impaired adipogenesis are thought to be the primary mechanisms for the link between epicardial fat and CVDs risk factors [21]. Therefore, a significant reduction in epicardial fat seems to be a logical goal for any obesity control approach. Emerging evidence highlighted that weight reduction is an effective approach for epicardial fat thickness reduction [22]. In the present study, we found that LSG led to a statistically significant reduction in the epicardial fat thickness twelve months after the operation. The extent of decrease in the epicardial fat thickness was more notable following LSG than conservative management.

In line with our findings, a previous retrospective study demonstrated a substantial decrease in the epicardial fat thickness following bariatric surgery; this reduction was linked to the degree of weight reduction [23]. A similar finding was noted in a more recent study byGaborit et al. [24]. In a meta-analysis reported by Rabkin and Campbell [25], patients who underwent bariatric surgery exhibited a considerable decline in epicardial fat thickness; this reduction was more prominent than the reduction achieved by diet alone. In addition, other studies showed the same findings [26–28].

The main limitation of the study is the lack of usage of magnetic resonance heart that may be more sensitive for assessing EFT. Nevertheless, a large sample size, prospective study design, with easier accessibility of use of echo are definite points of strength. Also, other studies are needed to compare if a particular type of bariatric surgery would be more efficient in this area. Besides, future carefully designed more extensive randomized-controlled studies with a more extended follow-up period are warranted to validate our results.

In conclusion, this work adds momentum to a growing literature by suggesting that LSG has a favourable impact on metabolic syndrome aspects, namely EFT, as it can decrease it considerably.

### Novelty of the study

To our knowledge, only a few studies addressed the topic of epicardial fat thickness after LSG. In addition, among the strengths of this study is the reasonable number of cases and the control group with a quite acceptable follow-up period. This point may make our work fairly unique as it is often difficult to get patients back for a second follow-up after such an interval.

Furthermore, this work points novel horizons into this field of research; and possibly attracts attention for this understudied issue, and this may provide new additions to armamentarium of the impact of bariatric surgery.

In other words, it is sensible to say that the current work could serve as a seminal study in the future, given the originality of the contribution and its clinically relevant nature. Most importantly, given the statistical results, the study underlines the fact that LSG has a measurable impact on epicardial fat thickness. Consequently, this shall reinforce the concept that the effects of LSG are primarily metabolic and physiological rather than merely anatomical/mechanistic.

## References

[1] Chooi YC, Ding C, Magkos F. The epidemiology of obesity. Metabolism. 2019; 92:6–10. 30253139 10.1016/j.metabol.2018.09.005

[2] Abdelaal M, Le Roux CW, Docherty NG. Morbidity and mortality associated with obesity. Ann Transl Med. 2017;5(7):161. 28480197 10.21037/atm.2017.03.107 PMC5401682

[3] Dixon JB. Obesity and diabetes: the impact of bariatric surgery on type-2 diabetes. World J Surg. 2009;33(10):2014–2021. 19421812 10.1007/s00268-009-0062-y

[4] Grundy SM, Hansen B, Smith SC, et al. Clinical management of metabolic syndrome: report of the American heart association/National heart, lung, and blood institute/American diabetes association conference on scientific issues related to management. Arterioscler Thromb Vasc Biol. 2004; 24(2):e19–e24. 14766740 10.1161/01.ATV.0000112379.88385.67

[5] Wadden TA, Sarwer DB, Fabricatore AN, et al. Psychosocial and behavioral status of patients undergoing bariatric surgery: what to expect before and after surgery. Med Clin North Am. 2007;91(3):451–469. 17509389 10.1016/j.mcna.2007.01.003

[6] Ruban A, Stoenchev K, Ashrafian H, et al. Current treatments for obesity. Clin Med (Lond)). 2019;19(3):205–212. 31092512 10.7861/clinmedicine.19-3-205 PMC6542229

[7] Grunvald E. Medical management of obesity: a comprehensive review. In: Clinical obstetrics and gynecology. Lippincott Williams and Wilkins, 2014. p. 465–484. 10.1097/GRF.0000000000000041 24979356

[8] Chang SH, Stoll CRT, Song J, et al. The effectiveness and risks of bariatric surgery: an updated systematic review and meta-analysis, 2003-2012. JAMA Surg. 2014;149(3):275–287. 24352617 10.1001/jamasurg.2013.3654 PMC3962512

[9] Lee SY, Lim CH, Pasupathy S, et al. Laparoscopic sleeve gastrectomy: a novel procedure for weight loss. Singapore Med J. 2011;52(11):794–800. 22173248

[10] Salman MA, Salman AA, Nafea MA, et al. Study of changes of obesity-related inflammatory cytokines after laparoscopic sleeve gastrectomy. ANZ J Surg. 2019;89(10):1265–1269. 31508889 10.1111/ans.15427

[11] Yano Y, Vongpatanasin W, Ayers C, et al. Regional fat distribution and blood pressure level and variability: the dallas heart study: fat distribution and blood pressure. Hypertens (Dallas, Tex 1979). 2016;68(3):576–583. 10.1161/HYPERTENSIONAHA.116.07876 PMC4982814 27432862

[12] Williams MJ, Hunter GR, Kekes-Szabo T, et al. Regional fat distribution in women and risk of cardiovascular disease. Am J Clin Nutr. 1997;65(3):855–860. 9062540 10.1093/ajcn/65.3.855

[13] Iacobellis G, Willens HJ. Echocardiographic epicardial fat: a review of research and clinical applications. J Am Soc Echocardiogr. 2009;22(12):1311–1319. 19944955 10.1016/j.echo.2009.10.013

[14] Bertaso AG, Bertol D, Duncan BB, et al. Epicardial fat: definition, measurements and systematic review of main outcomes. Arq Bras Cardiol. 2013;101(1):e18–e28. 23917514 10.5935/abc.20130138 PMC3998169

[15] Goel R, Alharthi M, Jiamsripong P, et al. Epicardial fat and its association with cardiovascular risk: a cross-sectional observational study. Hear Views. 2010;11(3):103. 10.4103/1995-705X.76801 PMC3089830 21577377

[16] Fried M, Yumuk V, Oppert JM, et al.; European Association for the Study of Obesity Obesity Management Task Force (EASO OMTF). Interdisciplinary European guidelines on metabolic and bariatric surgery. Obes Surg. 2014;24(1):42–55. 24081459 10.1007/s11695-013-1079-8

[17] Neeland IJ, Poirier P, Després JP. Cardiovascular and metabolic heterogeneity of obesity: clinical challenges and implications for management. Circulation. 2018;137(13):1391–1406. 29581366 10.1161/CIRCULATIONAHA.117.029617 PMC5875734

[18] Bays HE. Adiposopathy is "sick fat" a cardiovascular disease?. J Am Coll Cardiol. 2011;57(25):2461–2473. 21679848 10.1016/j.jacc.2011.02.038

[19] Chaowalit N, Somers VK, Pellikka PA, et al. Subepicardial adipose tissue and the presence and severity of coronary artery disease. Atherosclerosis. 2006;186(2):354–359. 16183065 10.1016/j.atherosclerosis.2005.08.004

[20] Sarin S, Wenger C, Marwaha A, et al. Clinical significance of epicardial fat measured using cardiac multislice computed tomography. Am J Cardiol. 2008;102(6):767–771. 18774004 10.1016/j.amjcard.2008.04.058

[21] Bays HE, González-Campoy JM, Bray GA, et al. Pathogenic potential of adipose tissue and metabolic consequences of adipocyte hypertrophy and increased visceral adiposity. Expert Rev Cardiovasc Ther. 2008;6(3):343–368. 18327995 10.1586/14779072.6.3.343

[22] Wu Y, Zhang A, Hamilton DJ, et al. Epicardial fat in the maintenance of cardiovascular health. Methodist Debakey Cardiovasc J. 2017;13(1):20–24. 28413578 10.14797/mdcj-13-1-20 PMC5385790

[23] Willens HJ, Byers P, Chirinos JA, et al. Effects of weight loss after bariatric surgery on epicardial fat measured using echocardiography. Am J Cardiol. 2007;99(9):1242–1245. 17478151 10.1016/j.amjcard.2006.12.042

[24] Gaborit B, Jacquier A, Kober F, et al. Effects of bariatric surgery on cardiac ectopic fat: Lesser decrease in epicardial fat compared to visceral fat loss and no change in myocardial triglyceride content. J Am Coll Cardiol. 2012;60(15):1381–1389. 22939560 10.1016/j.jacc.2012.06.016

[25] Rabkin SW, Campbell H. Comparison of reducing epicardial fat by exercise, diet or bariatric surgery weight loss strategies: A systematic review and meta-analysis. Obes Rev. 2015;16(5):406–415. 25753297 10.1111/obr.12270

[26] Iacobellis G, Singh N, Wharton S, et al. Substantial changes in epicardial fat thickness after weight loss in severely obese subjects. Obesity. 2008;16(7):1693–1697. 18451775 10.1038/oby.2008.251

[27] Cobos MS, Gomez CO, Estfanous N, et al. Bariatric surgery decreases pericardial fat and lowers the risk of developing coronary artery disease. J Am Coll Surg. 2018;227(4):S13.

[28] Van Schinkel LD, Sleddering MA, Lips MA, et al. Effects of bariatric surgery on pericardial ectopic fat depositions and cardiovascular function. Clin Endocrinol. 2014;81(5):689–695. 10.1111/cen.12402 24392723

